# The Home Hospitals Association

**Published:** 1913-03-22

**Authors:** 


### The Home Hospitals Association.

The thirty-fifth annual meeting of this Association was held on Monday, 17th inst. The Chairman, Sir Henry Burdett, said: The report this year is the most successful in the history of this Association, which was commenced in 1876, and was opened in the year 1880, and has for thirty-two years done an increasing and most valuable work. It is remarkable in the sense that it is the only hospital in this country which was established as a hospital upon a completely paying basis, with the object of proving that you could conduct a hospital as a business and make it a great success. I have a great deal to say to you to-day on other subjects, and all I need mention on this point to confirm the accuracy of my statement is that it has not only endured, but it has been rebuilt and completely re-equipped on the most modern basis without having any necessity to appeal to the public. Further, since that was done it has gradually repaid more than half the whole sum expended in the rebuilding, and in the course of the next few years the outstanding balance will be refunded and entirely wiped out. In saying this, you ought to understand that the object of the Home Hospitals Association was to provide for paying patients—that is to say, it had to secure the most efficient accommodation, and the best of surgical aid and medical treatment at a cost which should be less than the very high rates which were then charged for very indifferent accommodation, but at the same time inclusive and adequate. So that the whole aim and object of the Committee has been so to appraise the charges each year that they shall keep the institution financed, and shall protect the pockets, as much as possible, of the patients who have the good fortune to be attended in its wards. It has had a great reputation, and deservedly; for the efficiency of the work done here, which hygienically may be appraised from the fact, as I believe I am correct in saying, that there has never been a case of blood-poisoning from the day it was opened to the present time, a period of over thirty years, and that the rapidity of the recoveries, and the health and comfort of the patients, due to supremely efficient nursing, have been probably greater than anywhere else.

The Nucleus of a Large Pay Hospital.

Sir John Bland Sutton, F.R.C.S., proposed a vote of thanks to the Chairman for his kindness in presiding, and for the address he had given, which comprised such a valuable epitome of the Insurance Act problem. One never knew what would happen even to such an institution as this under the Act; and if this institution should eventually become the nucleus of a large pay hospital run for the benefit of patients under the insurance system, it would be a very excellent nucleus. He often laid awake at night wondering what the outcome of this great measure would be, but he had never had such a clear view of it as was afforded by Sir Henry Burdett's speech. Mr. Campbell seconded, and it was carried by acclamation.

